# Supplementary material for: Model-based and phylogenetically adjusted quantification of metabolic interaction between microbial species
Source: PLoS Comput Biol. 2020 Oct 30;16(10):e1007951. doi: 10.1371/journal.pcbi.1007951 (PMC7657538; doi:10.1371/journal.pcbi.1007951)
Supplement: Attachment — Submitted filename: responses.pdf [file pcbi.1007951.s009.pdf]

Reviewer 1:

1. Title. The current title is somewhat misleading. Unlike MinPath, a previous very impactful accomplishment of the corresponding author, the PhyloMint pipeline, as well as the article per se, does not operate with a concept of metabolic pathways (a biologically solid and practically useful paradigm of predominant and semi-isolated segments of metabolic networks). Instead, it relies on a far more ambitious but less granular/accurate (albeit computationally more attractive) concept of automated genome-scale metabolic models. It is desirable to duly reflect it in the title to avoid confusion.

   - Great point. We now replaced "Pathway" with "Model" in the title, so the new title is "Model-based and phylogenetically adjusted quantification of metabolic interaction between microbial species".

2. Supplementary data. They are mentioned several times in the MS (line 121, 162, 188, 194) and they are seemingly important but not properly organized and referred to. Instead of a generic umbrella reference in its current form ("details can be found in supplementary data"), which is not helpful at all, every reference should point to a specific supplementary table, graph or file.

   Thank you for your suggestions. We have since explicitly specified Supplementary Tables & Files accordingly within the manuscript.

3. A more important weakness of the paper is a lack of even one meaningful biological illustration of the main observations and conclusions. A very weak attempt to do so (p. 6, lines 177-183) is really not helpful.

   Thanks for the comment. We agree that the lack of a biological illustration was a weakness in this paper. We also agree that presenting a biological example will go a long way in exemplifying to the readers the biological observations and conclusions we mention in the text. We have since added Figure 5 to the manuscript which exemplifies an observed complementarity between *B. infantis* and *B. longum*, specifically a sialic acid metabolism pathway utilized in HMO metabolism.

   (a) An interesting observation about possible metabolic complementarity of distinct species of Bifidobacteria actually has merit, which should be discussed explicitly (and not via a reference to a paper, which is also not very helpful in that sense). Examples of metabolic complementarity could have included: (i) B2 auxotrophy, which is characteristic for most Bifidobacteria except B. infantis, one of the earliest colonizers of breast milk-fed infants; (ii) ability/inability to utilize HMOs also distinguishing otherwise closely related B. longum and B. infantis; (iii) extracellular degradation of fucosylated and sialilated HMOs, which is uniquely characteristic of B. bifidum and may comprise a mechanism of metabolic cooperation with co-colonizing B. infantis.

   Thank you for your insight in regards to *Bifidobacteria spp.* interactions, we have since added Figure 5 to exemplify metabolic features found within the GEM models of *B. infantis* and *B. longum* to illustrate that known metabolic interactions between these species had been captured by CarveMe. Additionally, we were able show how these interactions may influence the cooperative index.

   (b) Considering this (or another) example implicated by PhyloMInt at some level of biological/biochemical details would strongly impact the interest of biologists to the methodology and conclusions. In fact, it would be important to openly state whether indeed at least some (or even one) of the known distinctive metabolic features were indeed picked up by CarveMe tool.

   We were able show that CarveMe was able to accurately reconstruct metabolic pathways for sialic acid utilization, such as those mentioned by [1, 2, 3]. However, CarveMe failed to pick up specifically exoα-sialidase mediated degredation of sialylated HMO carbohydrates as mentioned in [4]. This is mainly due to CarveMe's dependency on BiGG Models as a

reference database, and consequently the lack of species/strain specific metabolic pathways. This limitation has since been explicitly highlighted in the results and discussion section. [see lines 211-216, and 279-289]

(c) Off note, the fact that the quality of the PhyloMInt analysis may be only as good as underlying metabolic reconstructions that are full of errors, deficiencies and inconsistencies (which authors should have admitted while providing rationale for their choice of CarveMe over other overpromising automated model generators), does not diminish the importance of the developed downstream approaches and tools.

We also noted that while the tractability of a fully automated system through the use of CarveMe may be questionable, various methods such as gap filling, manual curation, or even interchanging GENREs for GEM reconstructions remain viable options in lue of CarveMe. Additionally, we have since included in our discussion that the contribution of this work lies not in CarveMe's ability to fully reconstruct a metabolic model, but rather on the inclusion of phylogeny based correction of cooperative and competition indices. [see line 279-289]

4. In the same context: line 181- "...dominated by taxonomically similar genomes (Helicobacter, Collinsella, Lachnospiraceae, and Ruminococcus)." What was meant by this sentence? That Helicobacter is a close relative of Ruminococcus? Seriously? And if not, then what? And this is only one of the two sentences containing a reference to actual biology... The reader may lose all interest and confidence over vague statements like this one.

   Thank you for your comments. Indeed, the wording of this sentence may be a bit confusing. What was meant by this sentence was that besides Bifidobacteria, several other bacterial species formed sub-community modules highly uniform for their own Genus after correcting for phylogenetic distance; these included Helicobacter, Collinsella, Lachnospiraceae, and Ruminococcus species. Our intent was to highlight that other genus of bacteria formed taxonomically uniform sub-community modules, not that these genus are taxonomically similar. We have since reworded this sentence. [see lines 218-220]

5. Providing one or two relevant metabolic examples would also allow authors to illustrate for the readers the entire conceptual apparatus of source and sink metabolites, seed compound sets and so forth that are very relevant for understanding of their analysis of metabolic interactions in microbial communities.

   Thank you for your great suggestion. We do believe that the concept of Complementarity/Competition indices may confuse some readers, especially concepts such as source nodes, sink nodes, and seed set metabolites. We agree that the readers of this manuscript may also further benefit from examples to help further exemplify and conceptionalize the ideas around the methodologies proposed by Levy and Borenstein.

   We have since provided a metabolic example for sialic acid metabolism (Figure 5). This figure provides one example of metabolic interactions that may influence cooperative indices. However the linear nature of this example does not make a compelling example for conceptionalizing seed set metabolites and how that may influence the calculations of Complementarity/Competition indices. We have since included an additional figure in the methods section (Figure 6), while also detailing the calculation of Complementarity/Competition indices with this toy example. Rather than proposing a new toy example, we re-illustrated the example provided by Levy et al. (2015, ref 37) which exemplified the calculation of the Complementarity Index; here we add to their example by also exemplifying the calculation of the Competition Index using the same example.

Reviewer 2

1. I really like the directions the authors were taking, but feel the presented research is preliminary. More experiments are required to boost the rigor of this presentation. I also understand so far there

are no control datasets with known species interactions for a robust estimation of the prediction accuracy, so one might have to be creative to come up with some metrics.

Thank you for your suggestions. As you have mentioned, there lacks a control data set to be able to validate our conclusions. The lack of a gold standard/control dataset for species interactions remains a challenge not only for this study, but many others within the field. However, we believe that the necessity to correct for phylogeny remains grounded. The notion of utilizing phylogenetic information to correct for a given metric is not unique (elaborated in response R2Q3). As other have shown, phylogeny is often a confounding factor when comparing two related species, as related species share genetic information/traits which making the comparison between species no longer independent. While we cannot definitively validate the accuracy, we have shown that the complementarity and competition indicies share correlation with phylogenetic distance, and thus cannot be directly compared to one-another without first addressing their phylogenetic relationship. [See line 40-45]

In light of this, we do acknowledge that the lack of a control dataset is a valid weakness. We have expanded upon and addressed these concerns within the discussion. [See line 274-278]

2. While the source and sink nodes are necessary for metabolic network construction, the authors need to show that having incomplete genomes does not impact the resulting networks, not just the number of nodes. Moreover, Metagenome assembled genomes may not miss genes randomly, genes co-located in the same genomic region tend to be missed together (a binning problem).

   Thank you for your comments. We agree that it is important to show that the GENRE models preform well in the context of incomplete genomes. Additionally, we also agree that gene missing from assemblies is not a random process, and that genes missing are often co-located. To address this, we have since re-simulated incomplete genomes as described in the methods section [see lines 322-340]. Additionally, we have included several other matrices (i.e. Jaccard similarity of network edges and network nodes) to compare between models generated from complete genomes and simulated incomplete genomes (See revised Figure 1), as well as elaborated on the interpretation [see lines 116-136]. Furthermore, to better represent this data, analysis was re-scaled to percentage of CDSs rather than a discrete number of CDSs removed; this will aid in the evaluation as the MAGs included in this analysis consisted of MAGs with $> 80\%$ completeness.

3. The advantage of controlling phylogenetic relatedness was not clearly shown. Are there other independent evidence that may corroborate the statement that normalized indices are better than those without normalization?

   Thank you for your comments. Although we are the first one to raise the problem and propose a solution to decouple the metabolic interaction from phylogenetic relatedness, the thought of phylogenetic distance as a confounder is not a novel idea. This has long been proposed by systematists such as Felsenstein [5], who argued that since closely related species share genomic information, the direct comparison between two species cannot be independent. Since then, techniques and methods such as phylogenetic independent contrasts, phylogenetic comparitive methods, phylogenetic graphical lasso, and others, have been proposed to decouple the effect of phylogony. We have since expanded upon this in our introduction [see lines 40-45].

   Additionally, we have shown in Figure 2, the contrast between phylogenetic distance and the complementarity/competition indicies. As we can see in Figure 2A, if we take the highest indicies at phlogenetic distance 0 (~ 1.8) and compared them to indices at phylogenetic distance 2.5 (mean ~ 2), the extreme values at phylogenetic distance of 0 are no longer significant. This effect is similarly observed in Figure 2B. We believe that the need for phylogenetic correction is illustrated in Figure 2, but we do agree that additional elaboration may be necessary. We have since added additional text clarifying this argument.

4. In the method section, "Metabolic competition and complementarity indices", is the method the same as that presented in Levy and Borenstein (2013, ref 30)? If not, please list and justify the innovations here.

   The methodology to calculate the Metabolic Competition and Complementarity indices is the same as the method presented by Levy and Borenstein (2013, ref 30), as cited in lines 383 and 389. We do note that while testing NetCooperate from Levy et al. (2015, ref 37), we discovered that the NetCooperate module implementation erroneously discards certain cases of SCCs (where a smaller potential SCC lies within a larger SCC) when calculating the Metabolic Complementarity indices. We thus utilize our own implementation of calculating the Metabolic Competition and Complementarity indices following the formulation outlined by Levy and Borenstein (2013, ref 30). We did not see the need to reference this bug in the manuscript as we did not utilize the NetCooperate software, but rather, followed the Levy and Borenstein (2013, ref 30) description for the implementation. In respect to the Metabolic Competition and Complementarity indices proposed in Levy and Borenstein (2013, ref 30), we provide no new innovations to their proposed formulation.

5. In the method section, "Phylogenetic distance", it seems that the authors presented a novel method. Has it been compared to established method, such as gANI (PNAS 2005;102:2567–2572) or GTDB-tk (https://doi.org/10.1093/bioinformatics/btz848)?

   Phylogenetic distance employed here is based on the relative evolutionary distances of species pairs in accordance to an inferred phylogeny. We construct the phylogenetic tree, using a set of phylogenetic marker genes, similar to the approach in GTDB-tk, so technically the approach is not new. The difference is that GTDB-tk uses all species, but our tree focused on gut species. The reason that we use the evolutionary distance computed based on the tree (the phylogenetic distance between two nodes is the sum of the branch lengths between the nodes) instead of gANI is that computing gANI for all pairs of genomes is computationally extensive, and gANI works well only for pairs of more closely related genomes as identification of orthologous genes between remote genomes for gANI calculation might be nontrivial. We have now added more details in the corresponding section in the Method "Phylogenetic distance".

References

[1] Sela DA, Chapman J, Adeuya A, Kim JH, Chen F, Whitehead TR, et al. The genome sequence of Bifidobacterium longum subsp. infantis reveals adaptations for milk utilization within the infant microbiome. Proceedings of the National Academy of Sciences. 2008;105(48):18964–18969. doi:10.1073/pnas.0809584105.

[2] Egan M, Motherway MO, Ventura M, van Sinderen D. Metabolism of Sialic Acid by Bifidobacterium breve UCC2003. Applied and Environmental Microbiology. 2014;80(14):4414–4426. doi:10.1128/aem.01114-14.

[3] Sela DA, Mills DA. The marriage of nutrigenomics with the microbiome: the case of infant-associated bifidobacteria and milk. The American Journal of Clinical Nutrition. 2014;99(3):697S–703S. doi:10.3945/ajcn.113.071795.

[4] Sela DA, Li Y, Lerno L, Wu S, Marcobal AM, German JB, et al. An Infant-associated Bacterial Commensal Utilizes Breast Milk Sialyloligosaccharides. Journal of Biological Chemistry. 2011;286(14):11909–11918. doi:10.1074/jbc.m110.193359.

[5] Felsenstein J. Phylogenies and the comparative method. The American Naturalist. 1985;125(1):1–15.
